# Clinical outcomes of endoscopic mucosal resection for large superficial nonampullary duodenal epithelial tumor: a single-center study

**DOI:** 10.1097/MEG.0000000000002932

**Published:** 2025-01-31

**Authors:** Federico Barbaro, Luigi Giovanni Papparella, Michele Francesco Chiappetta, Tommaso Schepis, Rossella Maresca, Livio Enrico Del Vecchio, Cristina Ciuffini, Silvia Pecere, Lucio Petruzziello, Guido Costamagna, Cristiano Spada

**Affiliations:** a Digestive Endoscopy Unit, Fondazione Policlinico Universitario Agostino Gemelli IRCCS; b Center for Endoscopic Research Therapeutics and training (CERTT), Università Cattolica del Sacro Cuore; c Digestive Endoscopy, Ospedale Isola Tiberina – Gemelli Isola, Rome, Italy

**Keywords:** duodenum, mucosal resection, SNADETs, underwater

## Abstract

**Background and aim:**

Endoscopic mucosal resection (EMR) of superficial nonampullary duodenal epithelial tumors (SNADETs) is challenging, and to date, only a few studies assessed the clinical outcomes of EMR in the duodenum. The aim of this study was to evaluate the efficacy and safety of EMR for the treatment of SNADETs >10 mm.

**Patients and methods:**

This is a single-center retrospective study reporting data from a cohort of consecutive patients undergoing EMR of large (>1 cm) SNADETs between January 2017 and December 2021.

**Results:**

A total of 81 patients with 83 lesions underwent EMR (70 conventional EMR, 13 underwater EMR). The median size was 20 mm (range: 10–60 mm). The mean procedure time was 45 ± 30 min, and the en-bloc resection rate was 47%. In all patients, SNADETs were successfully removed (i.e. technical success). Delayed bleeding occurred in 5 (6%) of EMRs. One delayed perforation occurred, which was managed surgically. Recurrence rate was 20% with a median follow-up period of 20.5 months. Recurrence was detected at the first endoscopic follow-up in 88% of cases, and all recurrences were successfully treated endoscopically. Lesion size (*P* = 0.04), previous endoscopic resection (*P* = 0.05), and piecemeal resection (*P* = 0.05) were independent risk factors of local recurrence after EMR on multivariate-adjusted analysis.

**Conclusion:**

Large duodenal adenomas can be effectively managed by EMR. However, duodenal EMR of large lesions carries a significant risk of early recurrence, with a nonnegligible risk of adverse events. Lesion dimension, piecemeal resection, and previous endoscopic resection were associated with a higher recurrence rate. Close endoscopic follow-up is recommended given the high incidence of early recurrence, which can be successfully treated endoscopically.

## Introduction

Superficial nonampullary duodenal epithelial tumors (SNADETs) are relatively rare, with prevalence rates ranging from 0.03 to 0.4% in routine endoscopy [1–4]. Although SNADETs are less frequently observed compared with adenomas in the other areas of the gastrointestinal tract, their incidence has been gradually increasing in recent years [5]. Most of these lesions are incidentally diagnosed in patients undergoing routine gastroscopy for unrelated indications [6]. This upward trend in incidence is mainly due to better accuracy of gastroscopy, new endoscopic detection technologies, increased awareness of this disease by endoscopists, and, to a lesser extent, some environmental factors (e.g. smoking) [7,8]. Nonampullary duodenal adenomas, unlike other benign polyps, follow the adenoma-carcinoma sequence and have significant malignant potential, ranging from 30 to 85% [9–11]. Hence, surgical or endoscopic excision of sporadic duodenal adenoma is mandatory [12].

Pancreaticoduodenectomy (Whipple’s procedure) is the standard treatment for duodenal cancer. Nevertheless, surgery is associated with significant morbidity and mortality rates, ranging from 30 to 40% and 1 to 4%, respectively [13–16], and it is considered unsuitable for SNADETs [17]. Endoscopic resection (ER) represents an alternative treatment option for SNADETs, offering the advantage of organ preservation and, consequently, the preservation of the patient’s postoperative quality of life. According to European Society of Gastrointestinal Endoscopy (ESGE) recommendation, endoscopic mucosal resection (EMR) represents the first-line resection technique for nonmalignant nonampullary duodenal adenomas [18]. However, compared with EMR performed for similar-size lesions in other gastrointestinal tracts, duodenal EMR shows higher rates of both complications and local/residual recurrence rate [10,19–22]. Alternative EMR techniques, such as underwater EMR (u-EMR) [23–25] or cold snare EMR [26–28] have been proposed, yet these results are still emerging and often with conflicting results. The aim of this study is to assess the clinical outcomes of duodenal EMR in patients with SNADETs from a single-center patient cohort, investigating potential risk factors associated with the procedure.

## Materials and methods

### Design of the study and patients

This is a single-center, retrospective, observational study of consecutive patients prospectively enrolled who underwent duodenal EMR at a single tertiary referral center – Fondazione Policlinico Universitario Agostino Gemelli - IRCCS of Rome. For the purpose of the present study, data from a cohort of consecutive patients who underwent hot EMR for SNADETs ≥10 mm between January 2017 and December 2021 were extracted and analyzed. In the analysis, conventional EMR (c-EMR) and u-EMR were included. All patients signed written informed consent before the endoscopic procedure. Inclusion criteria were (1) patients with endoscopic diagnosis of SNADET (biopsy before treatment was not required for the diagnosis), (2) patients aged ≥18 years, and (3) at least one follow-up endoscopy. Exclusion criteria were (1) involvement of the ampulla of Vater, (2) patients diagnosed with familial adenomatous polyposis, (3) lesion size ≤10 mm, (4) endoscopic signs of infiltrating lesion (Paris III morphology [29]) at image-enhanced magnifying endoscopy and chromoendoscopy, (5) lesions resected by cold snare polypectomy (CSP), and (6) lesions resected by means of endoscopic submucosal dissection [endoscopic submucosal dissection (ESD)]. The following data were collected: age and gender of patients; size, morphology according to Paris Classification, and location of lesions; previous biopsies, previous resection attempts (i.e. manipulated lesion following unsuccessful attempt of ER); procedure details (technique used, technical success rate, en-bloc resection); complications (delayed bleeding, intraoperative perforation, and delayed perforation); pathological findings, need for surgery, and local recurrence during endoscopic follow-up. The study was conducted according to the guidelines of the Declaration of Helsinki and approved by the local ethics committee. Written informed consent was obtained from participants in the study.

### Endoscopic mucosal resection procedure

**Fig. 1.**
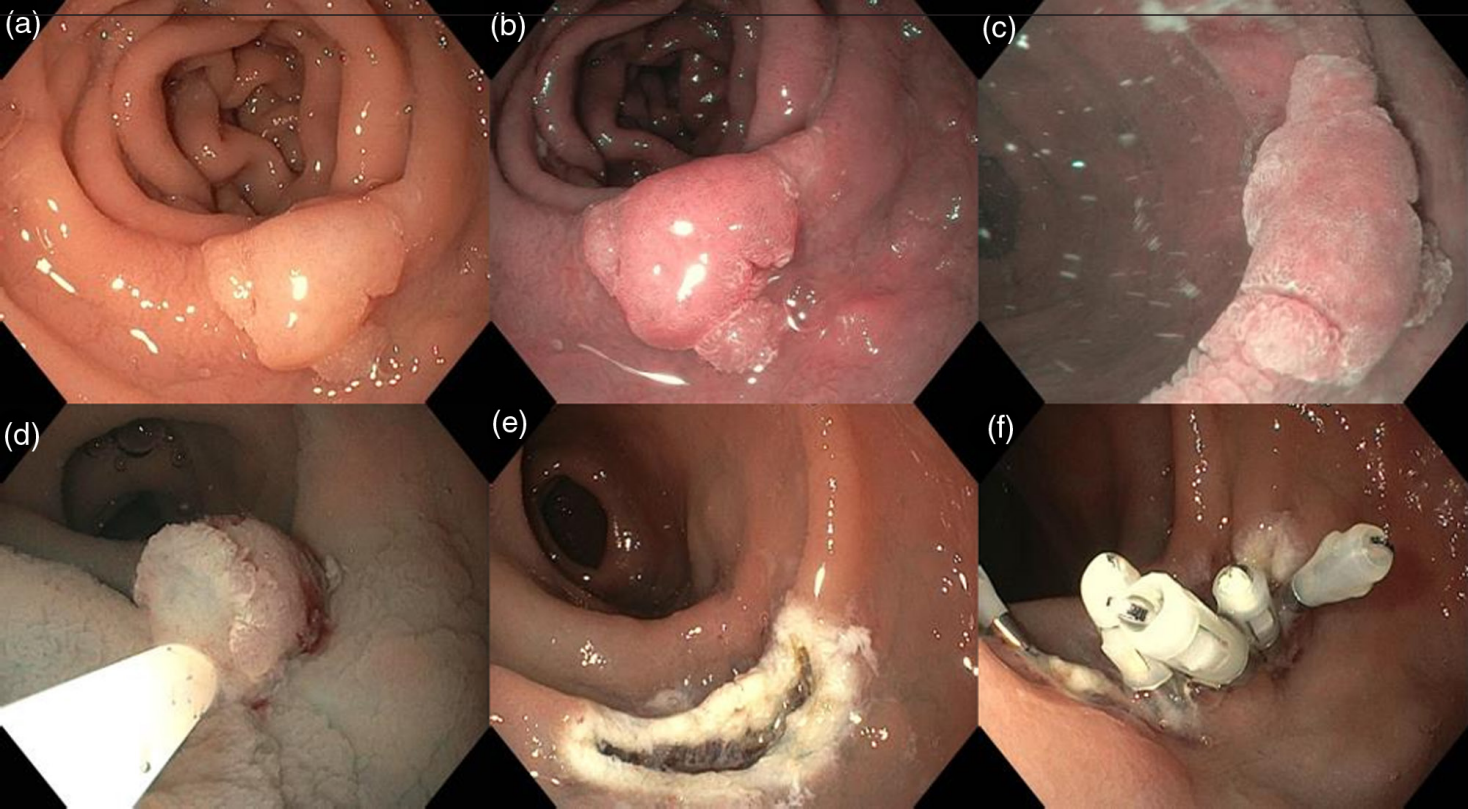
Underwater endoscopic mucosal resection (u-EMR) with partial submucosal injection for a superficial nonampullary duodenal epithelial tumor (SNADET). (a) A 15-mm flat elevated lesion is located on descending part of the duodenum. (b) Virtual Chromoendoscopy was used to clarify the border of the tumor. (c) Partial submucosal injection with saline and indigo carmine was performed on the distal edge of the lesion and the lumen was filled with saline. (d, e) The lesion was resected with a 15-mm hot snare in a single piece without any adverse events. (f) The mucosal defect was completely closed with five endoclips. Pathological findings revealed low-grade dysplasia adenoma with free horizontal and vertical margins.

EMR procedures were performed by four experienced endoscopists (F.B., G.L.P., L.P., and S.P.) with over 5 years of endoscopic procedure experience and more than 1000 ERs. Patients were treated as outpatients or inpatients at physician’s discretion. Deep sedation with propofol was adopted for outpatients while inpatients were treated under deep sedation or general anesthesia. Gastroscopes equipped with a water-jet system (GIF-H190 or GIF-EZ1500; Olympus, Tokyo, Japan) were used. Carbon dioxide was used for insufflation. If needed, a soft transparent hood was placed on the tip of the endoscope to improve visualization. Patients were treated with EMR by conventional technique (c-EMR) or underwater technique (u-EMR) at physician’s discretion. For c-EMR, after submucosal injection with a mixed solution of saline, indigo carmine, and epinephrine (dilution 1:100 000), the lesion was removed by means of 10–20-mm electrocautery snare (SnareMaster; Olympus, or Captivator; Boston Scientific, Marlborough, Massachusetts, USA). For u-EMR, after sucking out air, lumen was filled with saline or distilled water and the submerged lesion was resected by snaring (Fig. 1).

### Clinical outcomes and definitions

The primary evaluated outcome was the technical success rate. Technical success rate was defined as the rate of complete macroscopic resection with no visible residual adenomatous tissue observed endoscopically at the end of the procedure. The secondary outcomes were en-bloc resection rate, procedure time, complications (delayed bleeding, intraoperative perforation, and delayed perforation), and local recurrence. En-bloc resection was defined as complete removal of the lesion into one piece. For the assessment of c-EMR procedure time, submucosal injection was considered the beginning of the procedure time. For the assessment of u-EMR procedure time, the beginning of lumen filling with water or saline was considered the start of the procedure time. Both procedures were considered finished once all the mucosal fragments were removed. Time dedicated to hemostasis, managing complications, or prophylactic treatment of EMR margins were all included within the procedure time assessment. Delayed bleeding was defined as the presence of clinical signs of bleeding (such as hematemesis, melena, or hypotension) associated with a hemoglobin decrease ≥2 g/dl occurring up to 14 days after the procedure. Perforation was classified as intraoperative when a complete wall defect was observed during the procedure. Delayed perforation was categorized as the presence of extraluminal air and collections in the abdomen at postprocedural radiological findings. Biopsies on scars were performed when local recurrence was suspected following a thorough inspection of the resection site using white light and virtual chromoendoscopy. Local recurrence was defined as the presence of histologically confirmed adenomatous tissue at the prior resection site during endoscopic follow-up.

### Histopathologic analysis

Specimens were fixed in formalin, cut into 2-mm thick slices, and stained with hematoxylin and eosin. Lesions were categorized using the Vienna classification [30].

### Follow-up

Follow-up endoscopy was scheduled 3 and 12 months after EMR and annually thereafter for 5 years. When recurrence was detected, endoscopic retreatment was initially attempted in all cases. In case of recurrence, 3 months of surveillance was recommended until the resection site was free from recurrence.

### Statistical analysis

Continuous data were reported as median and range, whereas numbers and percentages were used for categorical variables. Mann–Whitney *U*-tests, Fisher’s tests, and chi-square test were used for the univariate analysis. A *P* value ≤0.05 was considered statistically significant. All analyses were conducted using SPSS version 25 for Windows (SPSS Inc., Chicago, Illinois, USA).

## Results

Eighty-one patients with a total of 83 SNADETs treated by EMR were included in the study (two patients had two synchronous SNADETs in different duodenal regions, which were removed in separate sessions). Median age of patients was 66 years (range, 25–85). Forty-nine percent of patients were male. Lesions had a median size of 20 mm (range, 10–60) and were in the bulb, in the second and third duodenal portion in 11, 76, and 13% of cases, respectively. SNADET macroscopic appearance was nonpolypoid in 59% of cases and polypoid in 41%. Using Paris classification, slightly elevated morphology (0-IIa) was the most frequent (43%), followed by sessile morphology (0-Is; 31%). Pedunculated (0-Ip) and semi-pedunculated (0-Isp) lesions represented 4 and 6% of cases, whereas mixed-type morphologies with a depressed (0-IIa + IIc) or sessile component (0-IIa + Is) characterized 6 and 5% of lesions. Flat lesions (0-IIb) represented 5% of the cases. Biopsies before EMR were performed in 45% of SNADETs and 10% of lesions had undergone a former unsuccessful attempt of complete ER. Most patients (93%) had been hospitalized to undergo the endoscopic procedure (Table 1).

**Table 1.** Patients and lesions characteristics

Characteristics	Total lesions (*n* = 83)
M/F, *n* (%)	49/34 (59/41)
Age (years), median (range)	66 (25–85)
Lesion size, mm, median (range)	20 (10–60)
Tumor location *n* (%)	
Bulb	9 (10.8)
Second portion	63 (75.9)
Third portion	11 (13.3)
Macroscopic type, *n* (%)	
Polypoid	34 (41.0)
Nonpolypoid	49 (59.0)
Previous biopsy	37 (44.6)
Previous failed attempt of resection, *n* (%)	8 (9.6)
Inpatient, *n* (%)	77 (92.8)
Paris classification, *n* (%)	
IIa	36 (43.4)
Is	26 (31.3)
IIa + IIc	5 (6.0)
Isp	5 (6.0)
IIa + Is	4 (4.8)
IIb	4 (4.8)
Ip	3 (3.6)

Patients were treated using conventional technique (c-EMR) in 84% of cases and underwater technique (u-EMR) in 16% of cases. Technical success was achieved in all the cases. The en-bloc resection rate was 47%. The median procedure time was 35 min (range, 12–181). Histopathologic examination reported low-grade dysplasia (LGD) in 71% of cases and high-grade neoplasia in 29% of cases [of which 92% high-grade dysplasia (HGD) and 8% carcinoma *in situ* (CIS)]. No intraprocedural complications occurred. A total of six patients (7%) suffered overall postprocedural complications. Delayed bleeding occurred in five patients (6%) and all cases were successfully managed endoscopically using clips and hemostatic forceps. The only late perforation (1%) occurred in a 53-year-old female with a 25-mm SNADET located at the inferior duodenal flexure that was removed by conventional piecemeal EMR. No intraprocedural perforation occurred. The mucosal defect was closed with four clips. Six hours after the procedure, the patient experienced abdominal pain and fever, with radiological signs of abdominal perforation. After 24 h, the patient underwent surgery. A 10-mm duodenal perforation was evident. A retroperitoneal toileting and duodenal surgical suturing were performed. Three weeks later, the patient was fully recovered and discharged. No procedure-related deaths occurred. Endoscopic follow-up was available in 100% of patients with a median follow-up period of 20.5 months (range, 3–55). Local recurrence was detected in 17 cases (20.4%), with 88% of these cases (15 cases) being diagnosed during the first follow-up endoscopy (Table 2).

**Table 2.** Outcomes and procedural details

Outcomes	Total lesions (*n* = 83)
Underwater resection, *n* (%)	13 (15.7)
Procedure time, min, median (range)	35 (12–181)
Technical success, *n* (%)	83 (100)
En-bloc resection, *n* (%)	39 (47.0)
Histopathology, *n* (%)	
Low-grade neoplasia	59 (71.1)
High-grade neoplasia (HGD/CIS)	24 (28.9)
Complications, *n* (%)	6 (7.2)
Delayed bleeding, *n* (%)	5 (6.0)
Perforation, *n* (%)	1 (1.2)
Surgery, *n* (%)	1 (1.2)
Follow-up, *n* (%)	83 (100)
Follow-up, months, median (range)	20.5 (3–55)
Recurrence, *n* (%)	17/83(20.5)
Recurrence at first FU	15/17 (88.2)

CIS, carcinoma *in situ*; FU, follow up; HGD, high-grade dysplasia.

Recurrences were all resected with cold snare technique. Pathology showed LGD at histopathological analysis in all cases. Patients with local recurrence had lesions of greater size (*P* = 0.04), longer procedural time (*P* = 0.05), and lower en-bloc resection rate (*P* = 0.0001) when compared with patients without local recurrence. Previous biopsies and previous attempts of ERs were statistically more frequent (*P* = 0.002 and *P* = 0.05, respectively) in the group with local recurrences (Tables 3 and 4).

**Table 3.** Characteristics of patients and lesions in recurrence and nonrecurrence groups

Characteristics	Recurrence, yes (*n* = 17)	Recurrence, no (*n* = 66)	*P*-value
M/F, *n* (%)	9/8 (52.9/47.1)	40/26 (61/39)	0.6
Age (years), median (range)	68 (48–78)	62 (25–85)	0.1
Lesion size (mm), median (range)	25 (10–60)	20 (10–60)	**0.04**
Tumor location, *n* (%)			
Bulb	1 (5.9)	8 (12.1)	0.3
Second portion	12 (70.6)	51 (77.3)	
Third portion	4 (23.5)	7 (10.6)	
Macroscopic type, *n* (%)			
Polypoid	4 (23)	30 (45)	0.08
Nonpolypoid	13 (77)	36 (55)	
Previous biopsy, *n* (%)	14 (82)	26 (39)	**0.002**
Previous endoscopic resection, *n* (%)	13 (76)	61 (92)	**0.05**
Inpatient, *n* (%)	17 (100)	60 (91)	0.2
Histopathology, *n* (%)			
Low-grade dysplasia	9 (53)	50 (76)	0.06
High-grade dysplasia/tumor in situ	8 (47)	14 (24)	

The values highlighted in bold indicate statistical significance.

**Table 4.** Outcomes in recurrence and nonrecurrence groups

Outcomes	Recurrence, yes (*n* = 17)	Recurrence, no (*n* = 66)	*P*-value
Underwater resection, *n* (%)	2 (11.8)	11 (16.7)	0.5
Procedure time (min), median (range)	55 (25–115)	33 (12–181)	**0.05**
En-bloc resection, *n* (%)	1 (5.9)	36 (54.5)	**0.0001**
Complications, *n* (%)	2 (11.8)	4 (6.1)	0.3
Delayed bleeding, *n* (%)	2 (11.8)	3 (4.5)	0.3
Perforation, *n* (%)	0	1 (1.5)	0.8
Surgery, *n* (%)	0	1 (1.5)	0.8

The values highlighted in bold indicate statistical significance.

Multivariate logistic regression analysis revealed that previous attempts of ER [odds ratio (OR), 7.4; 95% confidence interval (CI), 1.1–53.7; *P* = 0.04], piecemeal resection (OR, 9.6; 95% CI, 1.16–43.0; *P* = 0.04), and lesion size (1.04; 95% CI, 1.01–1.11; *P* = 0.05) were independent risk factors of local recurrence after EMR (Table 5).

**Table 5.** Univariate and multivariate analysis of predictors of local recurrence associated with endoscopic mucosal resection

Variable	Univariate analysis			Multivariate analysis		
	Odds ratio	95% CI	*P*-value	Odds ratio	95% CI	*P*-value
Age	1.05	0.97–1.11	0.17	1.07	0.89–1.15	0.21
Previous resection	**3.2**	**1.04–16.3**	**0.04**	**7.4**	**1.1–53.7**	**0.04**
Biopsy	**6.8**	**1.74–26.4**	**0.006**	2.3	0.8–14.0	0.12
Piecemeal resection	**6**	**1.2–69.2**	**0.01**	**9.6**	**1.16–43**	**0.04**
Lesion size	**1.05**	**1.01–1.10**	**0.01**	**1.04**	**1.01–1.11**	**0.05**
Histopathology (LGD/HGD-Tis)	2.8	0.90–8.75	0.08	1.7	0.34–8.72	0.5

CI, confidence interval; LGD, low-grade dysplasia; HGD, high-grade dysplasia; Tis, tumor in situ.

To assess clinical outcomes according to the type of procedure performed, we compared outcomes between c-EMR and u-EMR groups. SNADETs were treated by conventional technique (c-EMR) and underwater technique (u-EMR) in 84% (70/83) and 16% (13/83) of cases, respectively. Technical success rate of EMR was achieved in all cases. The en-bloc resection rate was 61.5% in the u-EMR group and 44.3% in the c-EMR group (*P* = 0.2). The median procedure time resulted higher in the u- EMR group (45 vs. 34 min, *P* = 0.001). Complications and recurrence rate were 7.7 and 15.4% in the u-EMR group and 7.1 and 21.4% in the c-EMR group, respectively, with no statistically significant difference between the two groups (*P* = 0.65; *P* = 0.47).

## Discussion

The results of this single-center study show that endoscopic treatment of large SNADETs is effective and safe. The reported 100% technical success rate and the relative low complications rate, even for large lesion, confirm that SNADETs should be primarily referred for ER. These results compare favorably with other recently published studies on EMR of similar-sized SNADETs, which reported technical success rates ranging from 90 to 96% [19,31]. Compared with other studies, our en-bloc resection rate (47%) results lower [19,31]. However, this can be attributed to the large size of the treated lesions and the exclusion of lesions under 10 mm from our series. Interestingly, none of our cases showed invasive cancer after resection. This could be the result of the endoscopist’s attitude to perform a meticulous evaluation of the mucosal pattern to diagnose signs predictive of submucosal infiltration and, therefore, to treat only lesions with no signs of invasion, given the high risk of incomplete ER and peri- and postprocedural complication rates.

It is noteworthy that in our patient cohort, of the 45% of lesions that had preresection histology (67% LGD, 33% HGD/CIS), 25% of LGDs were upgraded to HGDs and 37% of HGDs were downgraded to LGDs after histology on the resected specimen. These data are in agreement with ESGE recommendations [18] and confirm the limited usefulness of endoscopic duodenal biopsy sampling in the therapeutic management of duodenal lesions [3,32–35]. Furthermore, duodenal biopsies are associated with submucosal fibrosis that makes ER more challenging and increases the risk of perforation due to poor mucosal lifting [35].

The two major adverse events associated with EMR in the duodenum are bleeding and perforation. Previous data from Europe and Asia have shown an incidence of complication rates associated with c-EMR of 0–33% for delayed bleeding and 0–4% for perforation [6,31,36,37]. Similar rates of delayed bleeding and perforation have been associated with u-EMR, with no difference between c-EMR and u-EMR [25,38–43]. In our series, the overall adverse event rate was relatively low (7%) with five (6%) delayed bleeding and one (1%) delayed perforation. All delayed bleedings were successfully managed with endoscopic therapy while the delayed perforation required surgery. No procedure-related deaths occurred. Our subgroup analysis showed no statistically significant difference between c-EMR and u-EMR in terms of complication rates (7.1 vs. 7.7%, *P* = 0.65).

These results indicate a favorable safety profile for ER of duodenal lesions in expert hands, also considering that we included exclusively duodenal adenomas greater than 10 mm, of which 60% ≥20 mm and 29% ≥30 mm. However, adverse events can be hazardous and potentially fatal, and multidisciplinary management in a referral center with interventional radiologists and surgeons with experience in retroperitoneal surgery is essential. Substantial complication rates have been described especially after resection of large duodenal lesions. Fanning *et al*. reported 26.3% of major complications after EMR of giant nonampullary adenomas with a mean diameter of 40 mm, whereas Probst *et al*. showed 40% of delayed bleeding and 8% of perforation for nonampullary adenomas ≥30 mm. Recently, Probst *et al*. [36] demonstrated that lesion size and piecemeal resection are statistically predictive factors of any complication on adjusted analysis. Similarly, Maselli *et al*. [37] confirmed that increasing size of adenomas was an independent risk factor for perforation and bleeding on multivariate analysis, irrespective of endoscopic approach (EMR vs. ESD). In the present series, no predictors of adverse events were found at uni- and multivariate analyses, mainly due to the low incidence of adverse events as well as to the relatively small sample size of our cohort.

The rate of recurrence after duodenal EMR varies among published studies. Tomizawa *et al*. [6] in a large cohort study reported a local recurrence rate of 23%, whereas Klein *et al*. [19] showed a recurrence rate of 14.4% on the first surveillance endoscopy. In our study, a minimum of one endoscopic follow-up was available for 100% of patients. Local recurrence occurred in 20% of patients, and 88% of recurrences were diagnosed at the first endoscopic follow-up, scheduled at 3 months. Under this follow-up protocol, all the focal/residual recurrent lesions were successfully treated endoscopically, and no patients underwent surgery. In our series, we did not show any statistical significance difference in recurrence rates of u-EMR compared to c-EMR (15.4 vs. 21.4%, *P* = 0.47). This result aligns with data from a recent meta-analysis of six studies comparing u-EMR and c-EMR, which found no significant differences in recurrence rates. However, the current scientific evidence is primarily derived from retrospective comparative studies, and large prospective multicenter trials are still lacking. From our analysis, lesion size (*P* = 0.04), previous biopsy (*P* = 0.002), previous ER (*P* = 0.05), piecemeal resection (*P* = 0.0001), and a longer procedure time (*P* = 0.05) were associated with recurrence. While no association was found between recurrence and morphologic and histologic features as suggested by Singh *et al*., we have shown that previous resection attempts resulted as an independent predictor of recurrence (*P* = 0.04) on multivariate analysis, likely due to the difficulty of being curative in a high-grade fibrosis setting. In addition, we also showed that lesion size and piecemeal resection (which depends on lesion size) were predictive of recurrence, as already shown by Alexander *et al*. [44] and Kim *et al*. [45].

This study has several limitations. First, the retrospective, nonrandomized design may not exclude bias related to patient’s selection and to treatment protocols adopted for the resections. Second, duodenal polyps were managed using 2 different resection techniques (c-EMR and u-EMR) without well-defined protocols for the selection of the technique. Third, we have not included lesions removed by CSP or by ESD. Fourth, this is a single-center study. The Center involved in the study is a referral center with high endoscopy volumes. Therefore, the results of this study may not be directly translated to low-volume centers without a specific expertise in complex resections. Although the rate of local/residual recurrence is in accordance with previous studies, it cannot be excluded that the rate of recurrence or unknown complications are higher. Similarly, long-term recurrence is poorly evaluated in the present series and further studies addressing also long-term follow-up are needed to confirm the long-term rate of recurrence.

### Conclusion

Endoscopic mucosal resection of large (>10 mm), sporadic, nonampullary duodenal adenoma is effective. When compared with other endoscopic mucosal resections, the risk of adverse events is still higher suggesting that ER of large duodenal adenomas should be performed in referral centers. Previous attempts of ER, piecemeal resection, and lesion size were demonstrated to be independent risk factors of local recurrence after EMR.

## Acknowledgements

None.

### Conflicts of interest

There are no conflicts of interest.
